# Melanocytes Affect Nodal Expression and Signaling in Melanoma Cells: A Lesson from Pediatric Large Congenital Melanocytic Nevi

**DOI:** 10.3390/ijms17030418

**Published:** 2016-03-22

**Authors:** Naira V. Margaryan, Alina Gilgur, Elisabeth A. Seftor, Chad Purnell, Nicoleta C. Arva, Arun K. Gosain, Mary J. C. Hendrix, Luigi Strizzi

**Affiliations:** Stanley Manne Children’s Research Institute, Ann & Robert H. Lurie Children’s Hospital of Chicago, Northwestern University Feinberg School of Medicine, Chicago, IL 60611, USA; nmargaryan@luriechildrens.org (N.V.M.); agilgur@luriechildrens.org (A.G.); eseftor@luriechildrens.org (E.A.S.); chad-purnell@northwestern.edu (C.P.); narva@luriechildrens.org (N.C.A.); argosain@luriechildrens.org (A.K.G.); m-hendrix@northwestern.edu (M.J.C.H.)

**Keywords:** pediatric nevi, melanocytes, melanoma, melanosomes, anti-oxidants, Nodal

## Abstract

Expression of Nodal, a Transforming Growth Factor-beta (TGF-β) related growth factor, is associated with aggressive melanoma. Nodal expression in adult dysplastic nevi may predict the development of aggressive melanoma in some patients. A subset of pediatric patients diagnosed with giant or large congenital melanocytic nevi (LCMN) has shown increased risk for development of melanoma. Here, we investigate whether Nodal expression can help identify the rare cases of LCMN that develop melanoma and shed light on why the majority of these patients do not. Immunohistochemistry (IHC) staining results show varying degree of Nodal expression in pediatric dysplastic nevi and LCMN. Moreover, median scores from Nodal IHC expression analysis were not significantly different between these two groups. Additionally, none of the LCMN patients in this study developed melanoma, regardless of Nodal IHC levels. Co-culture experiments revealed reduced tumor growth and lower levels of Nodal and its signaling molecules P-SMAD2 and P-ERK1/2 when melanoma cells were grown *in vivo* or *in vitro* with normal melanocytes. The same was observed in melanoma cells cultured with melanocyte conditioned media containing pigmented melanocyte derived melanosomes (MDM). Since MDM contain molecules capable of inactivating radical oxygen species, to investigate potential anti-oxidant effect of MDM on Nodal expression and signaling in melanoma, melanoma cells were treated with either *N*-acetyl-l-cysteine (NAC), a component of the anti-oxidant glutathione or synthetic melanin, which in addition to providing pigmentation can also exert free radical scavenging activity. Melanoma cells treated with NAC or synthetic melanin showed reduced levels of Nodal, P-SMAD2 and P-ERK1/2 compared to untreated melanoma cells. Thus, the potential role for Nodal in melanoma development in LCMN is less evident than in adult dysplastic nevi possibly due to melanocyte cross-talk in LCMN capable of offsetting or delaying the pro-melanoma effects of Nodal via anti-oxidant effects of MDM.

## 1. Introduction

Normal melanocytic function determines skin pigmentation and protection from UV radiation, whereas dysfunction can lead to abnormal growth with the potential for malignant transformation to melanoma [1]. A major risk factor for melanoma development is the number and types of nevi caused by melanocytic proliferation [2]. Studies have shown how melanocytes affect the cutaneous microenvironment through molecular cross-talk mechanisms, mainly by means of secretory factors including pigment containing exosomes or melanosomes, growth factors and cytokines [3,4]. In addition, metabolic substrates like the amino acid tyrosine and by-products of synthesis and processing of hormonal precursors, such as dihydroxyphenylalanine (DOPA) can regulate melanin synthesis and underscore how alterations in nutritional and neuroendocrine processes can be implicated in affecting cellular and microenvironmental homeostasis of melanocytes [5,6].

Previous studies have reported the expression and activity of the TGF-β related growth factor Nodal and its association with aggressive advanced stage cancers, including those of the breast, prostate and melanoma [7]. Nodal is a developmental morphogen rarely detected in normal adult tissues and known to play a role in stem cell maintenance, regulation of organ development and positioning [8]. Nodal signaling typically occurs after binding to a receptor complex consisting of the EGF-like protein Cripto-1 and type I (ALK4/7) and type II (ActRIIB) Activin-like kinase receptors. This binding triggers phosphorylation of the Smad2/3/4 complex, which can then translocate to the nucleus and activate transcription of developmental pathway genes including autoinduction of Nodal itself [9]. Studies have also shown Notch4 activity regulating Nodal function during development [10] as well as expression in melanoma cells [11].

A recent report investigating the potential role of Nodal, Cripto-1 and Notch4 in adult melanocytic lesions of the skin showed that significantly high levels of Nodal were detected in dysplastic nevi excised from adult patients who subsequently developed melanoma, compared to the control group of dysplastic nevi excised from adult patients with no history of melanoma [12]. This observation suggested that Nodal expression in dysplastic nevi may have the potential as a prognostic biomarker for melanoma aggressiveness and play a potential biologic role during subsequent malignant transformation of these early melanocytic lesions. If confirmed, this finding would serve particularly useful in categorizing patients where a lack of reliable biomarkers capable of assessing the risk for melanoma development currently exists.

Approximately one in 20,000 newborns is diagnosed with large congenital melanocytic nevi (LCMN) [13]. Several definitions have been proposed for LCMN with most of the terms descriptively emphasizing the exquisitely large size of these lesions, such as congenital melanocytic nevi present at birth measuring 20 cm or more in diameter [14]. Development of melanoma is a rare complication of LCMN and macroscopic features, such as size of LCMN and number of satellite nevi, can help estimate the likelihood of melanomagenesis; however, currently there are no molecular markers that can more accurately assess risk for melanoma development in patients with LCMN. Thus, insight into why only a fraction of these patients with LCMN have the potential for development of melanoma and the ability to distinguish this subgroup are desired. The aim of this study was to evaluate Nodal expression in pediatric LCMN and correlate this with patient data, including melanoma history, to determine clinical relevancy of Nodal in LCMN patients at high risk for melanoma and explore specific LCMN characteristics that could negatively affect Nodal function and potential melanomagenesis in LCMN patients with low risk for melanoma.

## 2. Results and Discussion

**Figure 1:**
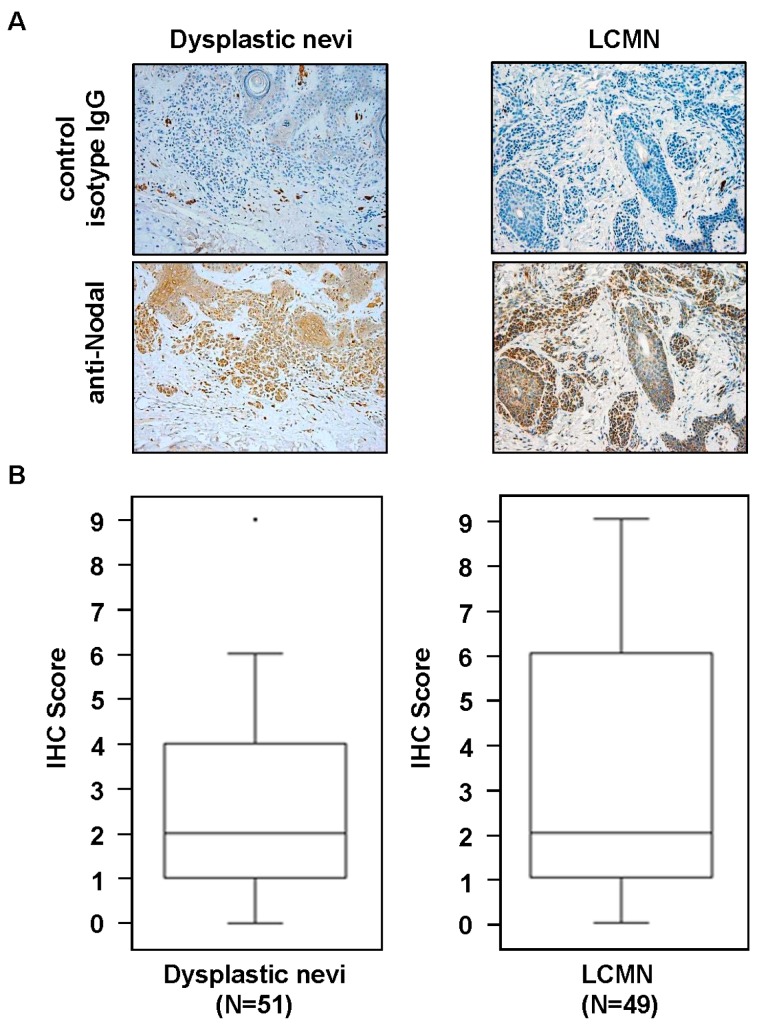
Immunohistochemistry results for Nodal staining. (**A**) Representative results show immunohistochemistry staining (brown) for Nodal in dysplastic nevi and LCMN from pediatric patients (20× original magnification); (**B**) Box plot representation shows that median staining scores for Nodal were similar between the two different groups analyzed.

**Figure 2:**
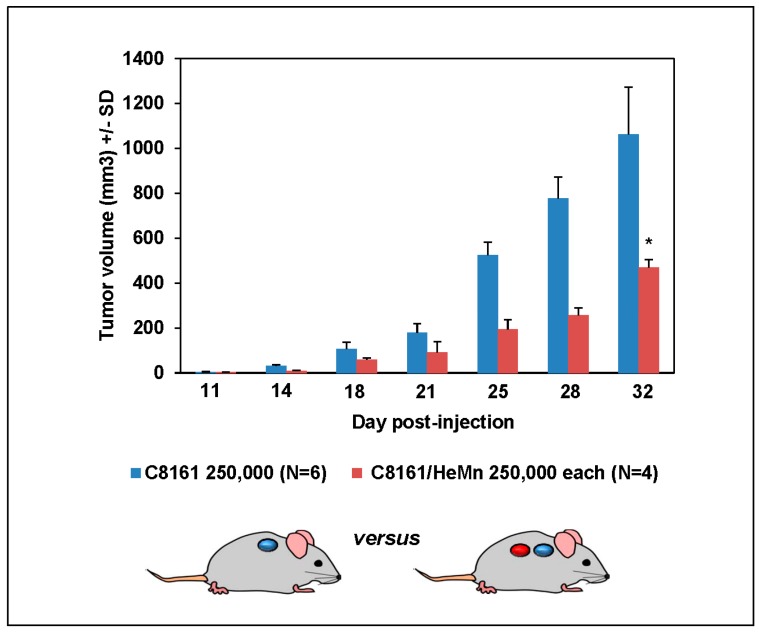
Growth of C8161 cells and HeMn melanocytes in Nude mice. At the end of the observation period, final tumor volume formed from the initial 250,000 human melanoma C8161 cells was significantly reduced when grown in close proximity to 250,000 HeMn melanocytes, compared to the final tumor volume of 250,000 C8161 allowed to grow alone (* *p* < 0.001).

Histological sections of skin biopsies from pediatric patients diagnosed with dysplastic nevi or LCMN were analyzed for Nodal expression by IHC. Although there were cases with high Nodal expression in dysplastic nevi and LCMN (Figure 1A), the overall median level of the IHC scores for Nodal expression was the same in both groups (dysplastic nevi (*n* = 51), median = 2 *versus* LCMN (*n* = 49), median = 2; *p* > 0.05 samples median test) (Figure 1B). Further analysis of Nodal expression in the LCMN cases did not reveal significant correlation with any of the available clinical data, such as gender, age at diagnosis or anatomical site of lesion. Since melanoma can develop in certain patients with LCMN [13], and because we have previously seen that high Nodal expression was detected in cases of adult dysplastic nevi that were excised years before the subsequent development of aggressive melanoma in the same patient [12], we reviewed the clinical data for history of melanoma in the LCMN patients and found that none of these patients had developed melanoma at the most recent follow-up, not even in those cases of LCMN with higher Nodal IHC staining scores. These observations appear to be at odds with the findings in adult dysplastic nevi. It is possible, therefore, that adult and pediatric melanocytic lesions are biologically different and this may affect the role of Nodal expression and function in these disparate lesions. Since one obvious difference is the extraordinarily high number of melanocytes present in LCMN, we investigated whether this could affect Nodal expression and/or function. To this end, an *in vivo* experiment was performed using the aggressive, high Nodal expressing human melanoma cell line C8161 and the normal human melanocyte cell line HeMn. Both cell lines were injected in Nude mice as a separate orthotopic bolus of 250,000 cells each in close proximity to determine how growth of each cell line would be affected by the presence of the other. Measurable tumor growth was detected only in the sites where C8161 cells were injected while the sites where HeMn cells were injected did not show any progressive growth. Most interestingly, at the end of the observation period, the C8161 cells initially injected in close proximity to HeMn cells in Nude mice showed significantly reduced final tumor volumes (470 ± 130 mm^3^; *n* = 4) compared to final tumor volumes of the control group of Nude mice bearing tumors formed by the C8161 cells injected in the absence of HeMn (1063 ± 146 mm^3^; *n* = 6) (*p* < 0.05) (Figure 2). Immunohistochemistry staining results of the tumors formed showed reduced intensity for Nodal, P-SMAD2 and P-ERK1/2 in the C8161 tumors grown in the presence of HeMn compared to C8161 tumors grown in the absence of HeMn (Supplementary Figure S1). The same effect of HeMn cells on final C8161 tumor volumes was observed when the number of HeMn cells injected in proximity to the bolus of C8161 cells was increased to 500,000 or 750,000 and when C8161 and HeMn cells were injected together as a (1:1) mixture of 250,000 cells each (final tumor volumes, respectively: 650.5 ± 192.6 mm^3^, *n* = 4; 360.1 ± 100 mm^3^, *n* = 4; 483.9 ± 233.6 mm^3^, *n* = 4), compared to final tumor volumes of the control group of Nude mice bearing tumors formed by C8161 cells injected alone (1264 ± 530 mm^3^; *n* = 4) (*p* < 0.05) (Supplementary Figure S2). These results suggest that possible cross-talk between HeMn melanocytes and C8161 melanoma cells could affect the growth of the melanoma xenograft in these Nude mice.

**Figure 3:**
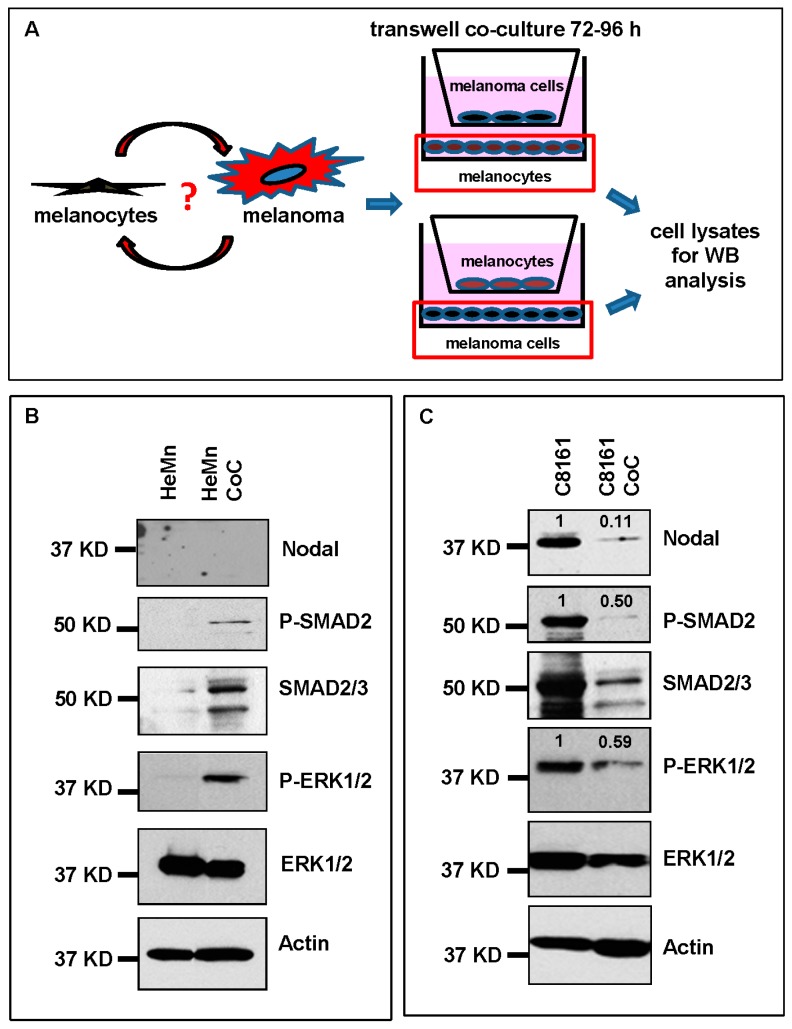
Co-culture (CoC) experiments. (**A**) Schematic diagram summarizing the co-culture experiments using HeMn melanocytes and C8161 melanoma cells. Lysates of cells growing on the bottom well (red square) were used for Western blotting; (**B**) Western blot results show increased expression of P-SMAD2 and P-ERK1/2 in HeMn melanocytes co-cultured with human C8161 melanoma cells compared to control HeMn cells culured alone. However, Nodal expression was not detected in HeMn cells grown alone or in co-culture with C8161 melanoma cells; and (**C**) Results from WB analysis show reduced levels of Nodal, P-SMAD2 and P-ERK1/2 in C8161 melanoma cells when co-cultured with HeMn melanocytes. Numbers above WB bands represent densitometric units, normalized to actin loading control, total SMAD2/3 or total ERK1/2, as appropriate, relative to control.

Morphologic and/or epigenetic changes in the expression of molecules involved in growth, proliferation and differentiation have been observed in normal melanocytes allowed to grow in the presence of or on substrates previously conditioned by keratinocytes, dermal fibroblasts, inflammatory cells and melanoma cells [15,16,17,18]. Nothing is known, however, how these conditions might affect Nodal expression and signaling. Understanding how microenvironmental factors may affect Nodal expression and function can shed light on the role Nodal plays in aggressive melanoma and suggest ways for increasing therapeutic effects of potential strategies to target Nodal [19,20]. To address this question, a transwell co-culture system (Figure 3A) was used to investigate *in vitro* the potential molecular effects of cross-talk between normal melanocytes and melanoma cells on Nodal expression and function. After 72 h of incubation, we found induction of Nodal signaling molecules P-SMAD2 and P-ERK1/2 in HeMn cells when co-cultured with C8161 cells compared to non-co-cultured HeMn cells (Figure 3B). Different molecular signaling events regulated by numerous melanoma derived soluble factors have the potential to induce SMAD2 and ERK activity in neighboring cells cells, however, the increased SMAD2 and ERK phosphorylation observed in HeMn cells co-cultured with the C8161 melanoma cells in this study does not seem to be Nodal dependent. This could be explained by the fact that melanocytes do not necessarily express the same receptor profile as melanoma cells that would otherwise allow them to respond to the exogenous Nodal from melanoma [21]. Nodal is capable of inducing its own expression using a feed-forward mechanism [8]; however, this did not appear to occur in the HeMn cells that were presumably stimulated with C8161 derived Nodal during the co-culture period (Figure 3B), again most likely because HeMn cells are poorly responsive to Nodal due to the lack of Nodal receptors. Nevertheless, Nodal is expressed at high levels in certain cases of adult dysplastic nevi [12,22] and, as shown for the first time in this study (Figure 1), in pediatric LCMN. It has been suggested that Nodal levels must reach a certain threshold for activation of its autoregulatory loop [23]. It is possible, therefore, that in cases of Nodal expressing melanocytic lesions, continued exposure to Nodal allowed for critical Nodal levels to be reached for autoinduction to occur, although participation of other unidentified cofactor(s) necessary for this process cannot be ruled out.

Interestingly, we found that expression of Nodal, P-SMAD2 and P-ERK1/2 were significantly reduced in C8161 cells when co-cultured for 72 h with normal HeMn human melanocytes (Figure 3C). This *in vitro* observation provided a potential explanation and molecular support for the reduced tumor volumes of C8161 when grown in proximity to HeMn cells observed *in vivo*. A similar trend showing reduced levels of Nodal, P-SMAD2 and P-ERK1/2 was also observed with a second melanoma cell line SK-MEL-28 co-cultured for 72 h with HeMn (Supplementary Figure S3A). These results suggest that soluble factors produced by melanocytes may negatively affect Nodal expression and signaling in melanoma cells.

**Figure 4:**
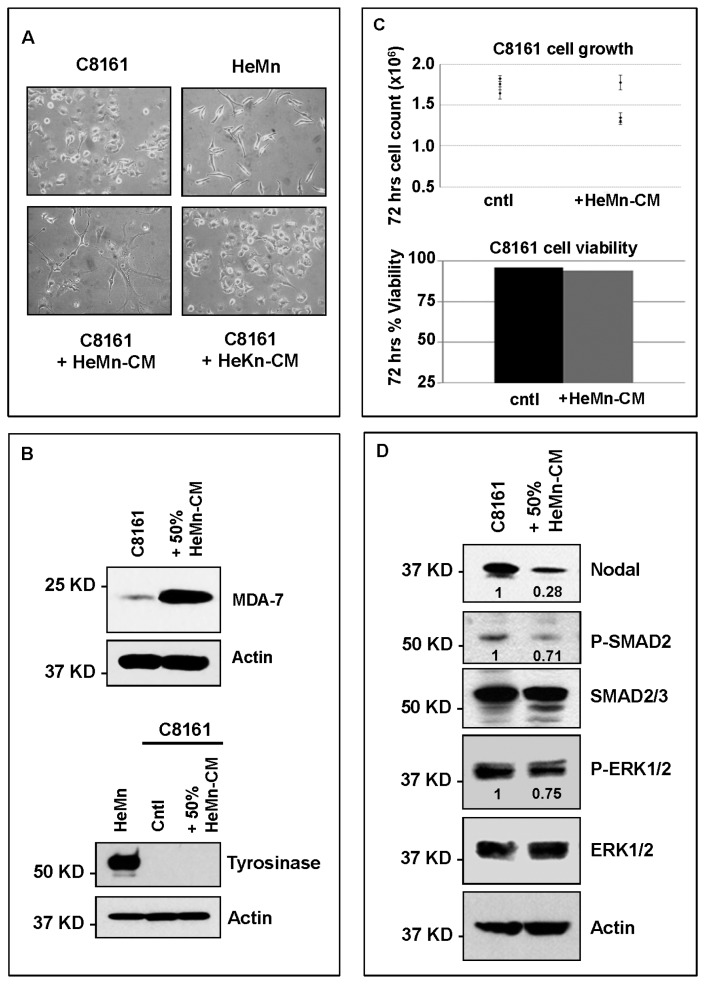
Effect of treatment of C8161 melanoma cells with HeMn melanocyte conditioned medium (HeMn-CM). (**A**) C8161 cells treated with HeMn-CM show morphologic changes such as dendritic shape with long cytoplasmic extensions reminiscent of melanocyte differentiation. (40× original magnification); (**B**) Although, Melanoma Differentiation Associated protein 7 (MDA-7) was detected in C8161 cells treated with HeMn-CM, the melanin synthesis associated enzyme tyrosinase was not; (**C**) C8161 cells treated with HeMn-CM showed similar proliferation rate and cell viability as non-treated control C8161 cells making potential toxic effect of HeMn-CM on C8161 unlikely; (**D**) Western blot analysis show that HeMn-CM treated C8161 express lower levels of Nodal, P-SMAD2 and P-ERK1/2 compared to untreated control C8161 cells. Numbers below WB bands represent densitometric units, normalized to actin loading control, total SMAD2/3 or total ERK1/2, as appropriate, relative to control.

To investigate the presence of soluble factors released by HeMn with potential effect on Nodal expression and signaling, we treated C8161 with 50% medium that was conditioned for 72–96 h by HeMn cells (HeMn-CM). After 72–96 h some of the C8161 cells in the HeMn-CM treatment group showed morphological changes, appearing larger than the untreated control C8161 cells and assuming a dentritic shape with cytoplasmic extensions reminiscent of normal melanocytes (Figure 4A). These morphological changes appeared to be specific to the HeMn-CM treatment since they were not observed when C8161 cells were treated with medium conditioned by HeKn human dermal keratinocytes (HeKn-CM) (Figure 4A). To determine whether HeMn-CM was inducing differentiation of C8161 cells to a more melanocyte-like phenotype, the HeMn-CM treated cells were analyzed for expression of the melanoma differentiation associated-7 protein (MDA**-**7), which has been shown to be induced during melanoma differentiation [24]. Results from WB analysis shows increased expression of MDA-7 protein in HeMn-CM treated C8161 cells compared to untreated control C8161 cells (Figure 4B). Previous studies have also shown that differentiating melanoma cells can increase the expression of the melanin producing enzyme, tyrosinase [25]. During the treatment period with HeMn-CM, however, C8161 cells did not show any difference in tyrosinase expression levels compared to untreated control cells (Figure 4B). Both induction of MDA-7 and the lack of tyrosinase expression in the HeMn-CM treated C8161 cells may be the result of toxicity of HeMn-CM on the melanoma cells. This is unlikely, however, since HeMn-CM treated C8161 cells did not show any significant difference in proliferation rate or cell viability during the treatment period compared to untreated C8161 cells (Figure 4C). Most importantly, WB analysis revealed reduced expression levels of Nodal, P-SMAD2 and P-ERK1/2 in HeMn-CM treated C8161 cells compared to untreated control cells (Figure 4D). A similar trend for reduction of Nodal, P-SMAD2 and P-ERK1/2 was observed in SK-MEL-28 human melanoma cells, also treated for 72 h with culture medium containing 50% HeMn-CM compared to untreated control SK-MEL-28 cells (Supplementary Figure S3B). Since HeMn cells appear to exert the same effect on both of the two different human melanoma cell lines tested, we continued our experiments with C8161 cells assuming that investigation of further effects of cross-talk between melanocytes and melanoma cells on Nodal would be more obvious in this cell line, which expresses higher levels of Nodal.

**Figure 5:**
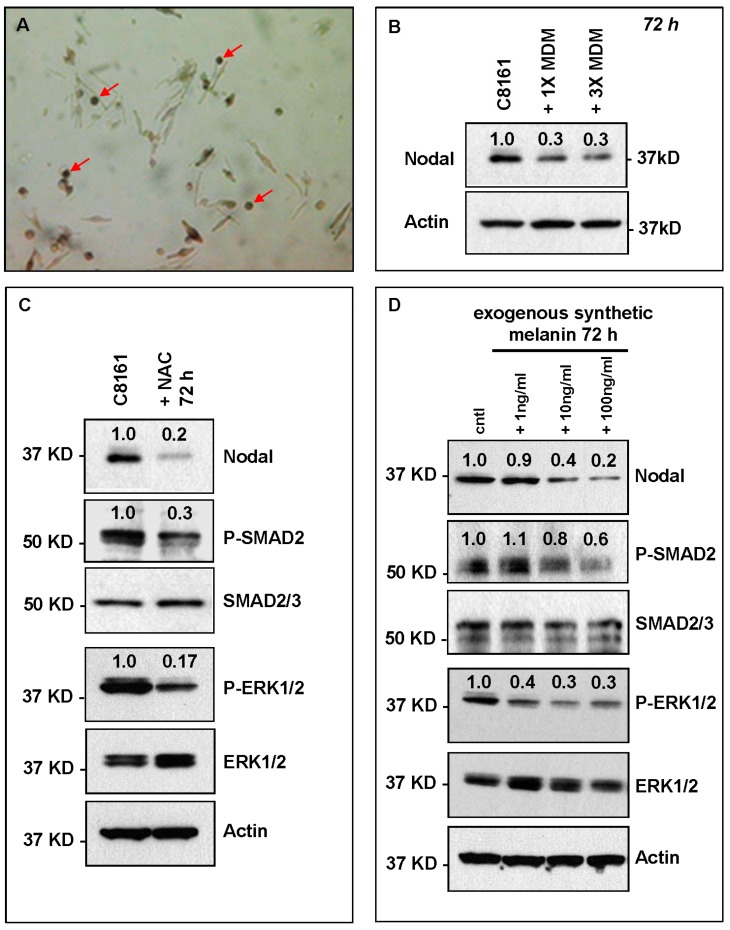
HeMn melanocyte derived melanosomes (MDM) affect Nodal expression and signaling components in C8161. (**A**) Microphotograph (40× original magnification) shows MDM containing globules (red arrows) produced by HeMn melanocytes; (**B**) C8161 melanoma cells treated with different concentrations of MDM collected from HeMn melanocytes showed reduction in Nodal expression compared to untreated control C8161 cells. To mimic the anti-oxidant effect of free radical scavengers contained in MDM, C8161 cells were treated with *N*-acetyl-l-cysteine (NAC) known to generate the anti-oxidant, glutathione; (**C**) Western Blot analysis show that treatment of C8161 melanoma cells with 5 mM NAC resulted in reduced levels of Nodal, P-SMAD and P-ERK1/2 compared to untreated control C8161 cells; (**D**) Treatment of C8161 cells with synthetic melanin, also known to have anti-oxidant properties, resulted in a dose-dependent reduction of Nodal, P-SMAD and P-ERK1/2 levels compared to untreated control C8161 cells. Numbers above WB bands represent densitometric units, normalized to actin loading control, total SMAD2/3 or total ERK1/2, as appropriate, relative to control.

Skin melanocytes are specialized pigment producing cells generally located at the dermal-epidermal junction responsible for skin pigmentation and extrapigmentary processes, such as antigen presentation and immune response [2]. In fact, breakdown of this specific immune regulatory response mechanism in melanocytes may explain, in part, how melanoma cells appear to evade host immune surveillance and anti-tumor activity [26]. In the skin, melanocytes produce globules with pigment containing melanosomes or melanocyte derived melanosomes (MDM) that can be released into the extracellular environment or transferred directly to keratinocytes and capable of diffusing across porous membranes in *in vitro* co-culture experiments [27,28]. Microscopic observation of the HeMn cells revealed small pigmented globules budding off the dendritic extensions of the melanocyte cell bodies and suspended in the culture medium (Figure 5A) similar to what has been described as MDM. We proceeded with the isolation of MDM by ultracentrifugation and resuspended the MDM containing pellets in culture media for treatment of C8161 cells. Results from Western blot analysis show reduction of Nodal protein level in C8161 cells treated for 72 h with 1× or 3× of MDM compared to untreated control C8161 cells (Figure 5B), suggesting that MDM and contents therein may negatively affect Nodal expression in melanoma cells. MDM contain many different types of biologically active molecules including those involved in free radical scavenger (FRS) activity [29]. FRS-related molecules are important for inactivating potentially toxic reactive oxygen species, mostly dopamine derived by products that are formed during the synthesis of melanin [2]. In fact, anti-oxidant activity has been shown to promote survival of melanocytes and also delay the onset of melanoma *in vivo* [30]. To determine whether these anti-oxidant effects capable of negatively affecting melanoma could involve regulation of Nodal expression and signaling, we treated C8161 cells with *N*-acetyl-l-cysteine (NAC), which provides the necessary amino acid l-Cysteine during generation of glutathione that exerts anti-oxidant effect, thus mimicking FRS activity of MDM. Western blot analysis of lysates from the NAC treated C8161 cells showed again reduced levels of Nodal, P-SMAD2 and P-ERK1/2 compared to untreated C8161 control cells (Figure 5C). Melanin is also a major component of MDM, and in addition to its role during pigmentation, can exert antioxidant effects [31]. Therefore, we treated C8161 cells with increasing concentrations of cell culture compatible synthetic melanin. C8161 cells treated with exogenous synthetic melanin showed a dose-dependent reduction of Nodal, P-SMAD2 and P-ERK1/2 compared to untreated control C8161 cells (Figure 5D). Hypoxia has been shown to positively regulate Nodal levels in cancer cells [32], therefore, oxidative stress and free radical production associated with hypoxic conditions [33] could potentially induce Nodal expression in affected cells and tissues. Thus, our observations suggest that one way melanocytes can negatively affect Nodal expression and signaling leading to reduction in growth of Nodal-dependent melanoma cells is by reducing the stimulatory effects that oxidative stress may exert on Nodal expression via anti-oxidant properties of MDM components.

As Nodal has been shown to affect melanoma aggressiveness and metastasis, and may potentially participate in creating the conditions that would favor transformation of dysplastic nevi to melanoma in the adult population, the results from this study show that this role could be affected by melanocyte activity. In fact, in pediatric LCMN, pigmented skin lesions with densely populated melanocytes covering a relatively large surface area, the potential role for Nodal in melanoma development does not appear to be as evident as observed in adult dysplastic melanocytic lesions. Our data show that one way in which the abundance of melanocytes may exert control over Nodal expression and signaling is through the anti-oxidant effects of FRS-related molecules contained within MDM. Perhaps the breakdown in this type of MDM-dependent regulatory cross-talk between normal melanocytes and melanocytes that have initiated cellular changes, could lead to both increased sensitivity towards Nodal function and chances of malignant transformation. In fact, in pediatric LCMN Nodal expression levels are significantly lower than the levels generally detected in the melanocytic lesions in adults [12] and do not correlate with subsequent development of melanoma, as seen in the high Nodal expressing adult dysplastic nevi [12].

Melanin synthesized by melanocytes is important for protecting the skin from chemicals, toxins, heavy metals and damaging radiation from the sun; it is evident therefore, that abnormalities in these melanin producing cells could lead to increased incidence of pigment disorders and malignancies of the skin including melanoma [1]. Thus, the role for Nodal during melanoma development could become more relevant later in life, when opportunity for altered melanocyte function leading to inefficient melanin production, coupled with increased exposure to and accumulation of the effects of potentially damaging environmental factors including UV radiation come into play. Increased levels of reactive oxygen species that can occur during senescence has been shown to be involved with development of melanoma [34]. It is possible, therefore, that the establishment of this oxidative environment, which appears to be kept in check earlier in life by anti-oxidants, such as those contained in MDM, could contribute to activation of pro-melanoma factors, such as Nodal. Interestingly, this age-associated role for Nodal in cancer has been suggested in glioblastoma where Nodal function appears to play a less obvious role in young pediatric glioblastoma patients [35] compared to the role described for Nodal during induction of aggressiveness in adult glioblastomas [36]. Further studies comparing the same types of malignancies in pediatric and adult patients will be needed to confirm the effect of patient age on the role for Nodal in cancer.

## 3. Materials and Methods

### 3.1. Patient Samples and Immunohistochemistry

Histological slides with sections of patient skin biopsies containing lesions diagnosed as dysplastic (atypical) nevi or LCMN were purchased from the Pathology Core at Lurie Children’s Hospital of Chicago, IL, USA. All slides were devoid of any patient identifier in accordance with Institutional Review Board (IRB) approved study protocol (IRB ID: 2014-15807). The tissue sections were processed for immunohistochemistry (IHC) as previously described [12]. Briefly, following antigen retrieval and blocking steps, sections were incubated in primary antibody for 60 min, followed by species appropriate biotinylated secondary antibodies (Biocare Medical, Concord, CA, USA), and then streptavidin peroxidase (Thermo Scientific Lab Vision, TS125HR, Fremont, CA, USA). Stain was developed with 3,3′-diaminobenzidine substrate (Thermo Scientific Lab Vision, TA125HDX) and sections were counterstained with hematoxylin (Biocare Medical, NM-HEM). Specifically, for IHC on patient tissue sections mouse monoclonal anti-Nodal (Abcam, ab55676, 1:200, Cambridge, MA, USA) was used as primary antibody. For IHC on mouse xenograft tissue sections, goat anti-Nodal (Lifespan Biosciences, LS-B3955, 1:100, Seattle, WA, USA), rabbit anti-P-SMAD2 (Cell Signaling, 3101, 1:500, Danvers, MA, USA) and rabbit anti-p44/42 MAPK (ERK1/2) (Cell Signaling, 4370, 1:400) were used as primary antibodies. As a negative control, adjacent serial sections were incubated with species appropriate irrelevant IgG (Jackson ImmunoResearch Labs, West Grove, PA, USA). The quality and intensity of staining were analyzed and scored at low power and high power in order to calculate an Index Score (IS), as previously described [22]. Median sample test and Student’s *t* test were used for statistical analysis of the IHC scoring and tumor volume data, respectively. *p*-Value < 0.05 was considered statistically significant.

### 3.2. Cell Cultures

The normal human melanocyte cell line HeMn-LP (HeMn) (ThermoFisher Scientific, Waltham, MA, USA) and human melanoma cells C8161 (University of Arizona, Tuscon, AZ, USA) and SK-MEL-28, ATCC) were used. Cell lines were authenticated by short tandem repeat genotyping at Lurie Children's Hospital of Chicago (Chicago, IL, USA). All cell lines were routinely tested for mycoplasma contamination using the Mycoplasma PCR ELISA Kit (Roche, 11663925910, Indianapolis, IN, USA) and maintained as previously described [11,19]. Culture media conditioned by HeMn for 72 h (HeMn-CM) was collected for use in subsequent treatment experiments with melanoma cells. Co-culture experiments were carried out using transwell system (Corning, 3460, Glendale, AZ, USA) and following manufactures instructions. This transwell system consists of small wells containing porous membranes on the bottom surface that are inserted into larger wells. The pores on the membrane are small enough to maintain physical separation of the two different cell types but allows for exchange of secreted factors including melanosomes [27]. At the end of the co-culture period, protein lysates were obtained from the cells grown on the bottom wells and analyzed by WB. Pigment globules containing melanosomes secreted by melanocytes were collected by ultracentrifugation, as previously described [27]. Pellets were resuspended in culture media to obtain 1× or 10× suspensions and used to treat melanoma cells. To investigate the role of FRS components of MDM on Nodal expression and signaling, C8161 cells were treated with 5 mM NAC, which was prepared as previously described [29]. C8161 cells were also treated with cell culture suitable synthetic melanin (Sigma, M0418, Saint Louis, MO, USA), which was prepared according to manufacturer instructions.

### 3.3. Flow Cytometry

Cell viability and proliferation assays were evaluated on a Guava easyCyte HT System Flow Cytometer (Millipore, Billerica, MA, USA) using ViaCount reagent (4000-0040) for analysis of viability and cell number and NEXIN reagent (4000-0450) for analysis of early and late apoptotic cells according to the manufacturer's instructions (Millipore). Parameters were set using untreated cells and analyzed in triplicate.

### 3.4. Cell Lysis and Western Blotting

Whole cell lysates were prepared in 25 mmol/L Tris pH 7.4, 0.5 mmol/L EDTA, 5% glycerol, 1% SDS and 1× Complete Mini protease inhibitors (Roche, 11836153001). Protein concentrations for lysates were determined by BCA assay (Thermo Scientific, PI23225) and diluted in Laemmli Sample Buffer (BioRad, 161-0737, Hercules, CA, USA) supplemented with β-mercaptoethanol and boiled for 10 min at 95 °C. 12% SDS-PAGE with 4% stacking gels were used to resolve lysates, with 10 μg of protein loaded per lane. Proteins were transferred to polyvinylidene difluoride (PVDF) membranes (BioRad, 162-0184) and identified using the appropriate primary and secondary antibodies + HRP with chemiluminescence detection. The following primary antibodies were used: mouse anti-tyrosinase (Abcam; ab738); rabbit anti-MDA-7 (Abcam; ab115207); rabbit anti-Nodal (Santa Cruz Biotechnology, H-110, Dallas, TX, USA); rabbit anti-P-SMAD2 (Life Technologies, 44-244G; Grand Island, NY, USA); rabbit anti-Smad2/3 (Millipore, 07-408, Lake Placid, NY, USA); rabbit anti-P-p44/42 MAPK (P-ERK1/2) (Cell Signaling, 9101S); rabbit anti-p44/24 MAPK (ERK1/2) (Life Technologies, 44-654-G); mouse anti-actin (Millipore, MAB1501; Millipore, Temecula, CA, USA). Densitometric analysis of WB bands, normalized to respective loading controls was performed and OD units relative to control reported where appropriate.

### 3.5. Animal Experiments

For *in vivo* experiments different groups of 6-week old virgin female Nude mice were injected orthotopically with approximately 250,000 of HeMn cells within 1 cm of 250,000 C8161 or SK-MEL-28 human melanoma cells also injected orthotopically. In another experiment, 250,000, 500,000 or 750,000 HeMn cells were injected orthotopically within 1 cm of 250,000 Nodal-expressing C8161 also injected orthotopically. Another set of mice were injected orthotopically with a 1:1 mixture of 250,000 HeMn cells and 250,000 C8161 cells. Control groups consisted of mice receiving only HeMn cells or only C8161 cells. As previously described [16], 10 days post-injection, measurements were taken twice per week over three weeks using appropriate calipers and tumor volumes calculated (*V* = π/6(*L* × *W* × *H*)). All animals were housed in the fully accredited Stanley Manne Children’s Research Institute’s animal facility and all experiments performed in accordance with the Stanley Manne Children’s Research Institute’s Institutional Animal Care and Use Committee (IACUC), Chicago, IL approved protocol (IACUC protocol ID: 14-014.0; approval 3 November 2014).

## 4. Conclusions

Nodal expression has been shown to be associated with aggressiveness in human cancer. In melanoma, Nodal appears to play a role in tumor cell plasticity and metastatic spread. Recently, high levels of Nodal have been detected in adult dysplastic nevi excised form patients that subsequently developed melanoma suggesting that Nodal expression could be used as a biomarker for melanomagenesis in these patients. This would improve our ability to identify patients in categories at risk for melanoma like pediatric patients with LCMN. In this study, however, this role for Nodal to predict melanoma in pediatric LCMN patients was not observed. Moreover, we show that melanocytic activity via anti-oxidant components of MDM is capable of mitigating Nodal expression and signaling in melanoma cells and could explain how the large numbers of melanocytes like those present in LCMN may control potential Nodal dependent pro-melanoma activity. Thus, patient age and/or microenvironmental factors, such as number and function of melanocytes may be important factors capable of influencing expression and role of Nodal in melanoma. With recent preliminary studies showing the potential benefit of anti-Nodal therapy in melanoma, the timing of such therapy with respect to patient age or degree of skin pigmentation becomes important when selecting conditions for optimal Nodal target expression for maximum therapeutic effect.

## Supplementary Materials

Supplementary materials can be found at http://www.mdpi.com/1422-0067/17/3/418/s1.
