# Supplementary material for: Melanocytes Affect Nodal Expression and Signaling in Melanoma Cells: A Lesson from Pediatric Large Congenital Melanocytic Nevi
Source: Int J Mol Sci. 2016 Mar 22;17(3):418. doi: 10.3390/ijms17030418 (PMC4813269; doi:10.3390/ijms17030418)
Supplement: Supplementary file 1 [file ijms-17-00418-s001.pdf]

Supplementary Material: Melanocytes Affect Nodal Expression and Signaling in Melanoma Cells: A Lesson from Pediatric Large Congenital Melanocytic Nevi

Naira V. Margaryan, Alina Gilgur, Elisabeth A. Seftor, Chad Purnell, Nicoleta C. Arva, Arun K. Gosain, Mary J. C. Hendrix and Luigi Strizzi

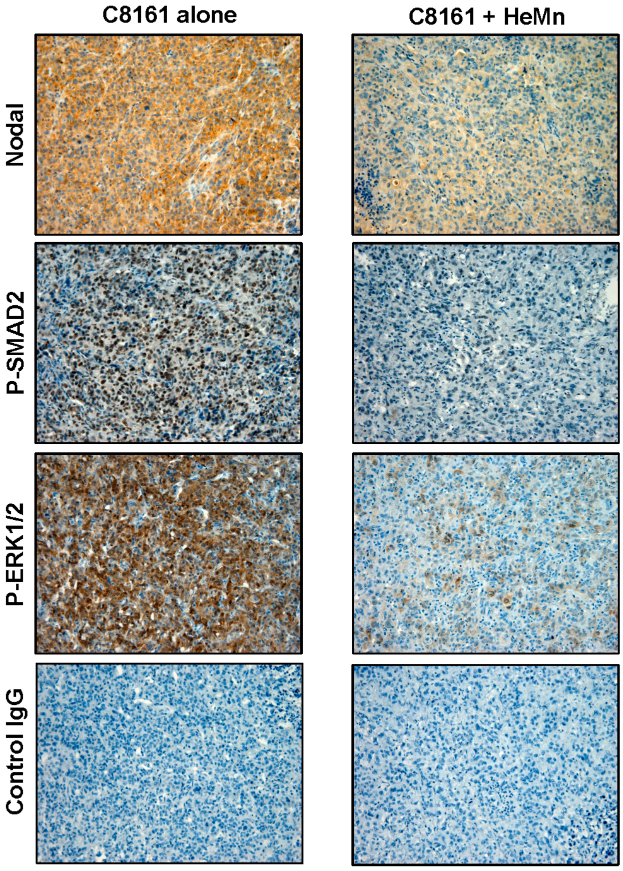

Figure S1. Immunohistochemistry staining of xenograft tumors. Results from immunohistochemistry show decreased staining intensity for Nodal, P-SMAD2 and P-ERK1/2 in tissue sections from tumors formed by C8161 melanoma cells grown in the presence of HeMn melanocytes (C8161 + HeMn) compared to the intensity of staining in for each marker in sections from tumors formed by C8161 grown in the absence of HeMn (C8161 alone). Control IgG shows representative images from staining with species specific irrelevant IgG (20× original magnification).

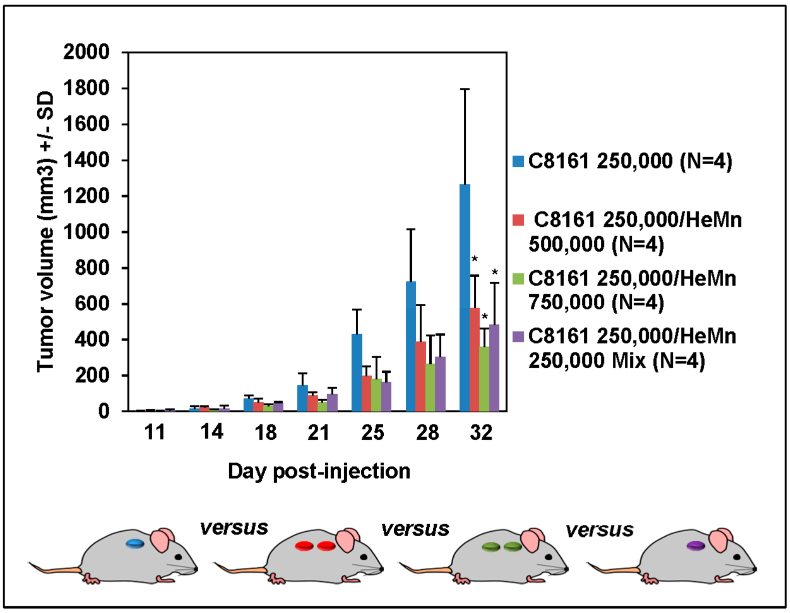

Figure S2. Growth of C8161 Nude mouse xenograft in the presence or absence of HeMn melanocytes. At the end of the observation period, tumor volumes formed from the initial injection of 250,000 human melanoma C8161 cells were significantly reduced when grown in close proximity to initial injections of 500,000 or 750,000 HeMn melanocytes compared to final tumor volume of initial injection of 250,000 C8161 cells grown alone. A mixture of 250,000 each of C8161 cells and HeMn melanocytes also resulted in significant final tumor volume reduction compared to tumor volume of C8161 cells grown alone (* $p < 0.05$).

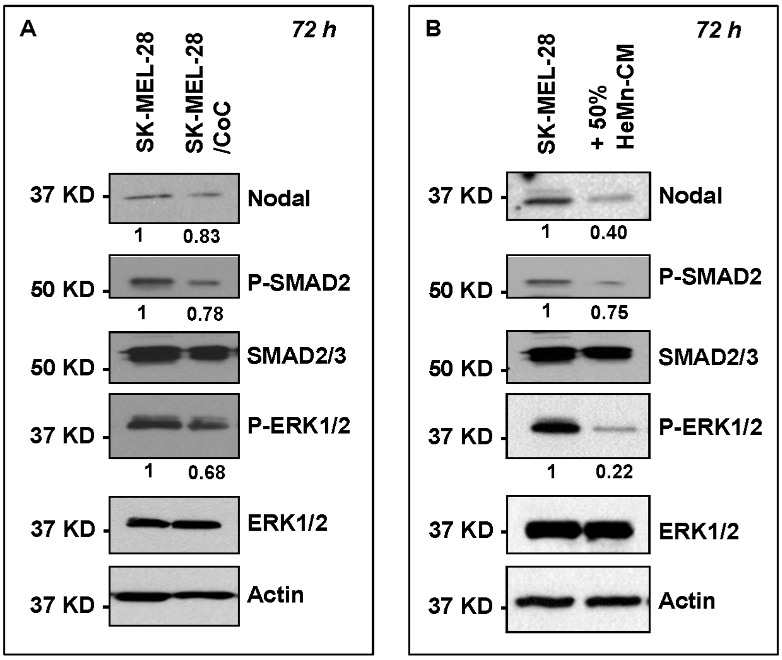

Figure S3. Western blot results from co-culture experiments and treatment with HeMn conditioned medium (HeMn-CM) in SK-MEL-28 melanoma cells. As observed with C8161 cells SK-MEL-28 melanoma cells show lower levels of Nodal, P-SMAD2 and P-ERK1/2 when co-cultured with HeMn melanocytes (**A**); or treated with HeMn-CM (**B**). Numbers below WB bands represent densitometric units, normalized to actin loading control, total SMAD2/3 or total ERK1/2, as appropriate, relative to control.
